# Giant Cell Arteritis Manifesting as Area Postrema Syndrome: A Case Report

**DOI:** 10.7759/cureus.93470

**Published:** 2025-09-29

**Authors:** Caitlin Courtney, Catherine Franconi, Ferry Dharsono, David Prentice

**Affiliations:** 1 Neurology, Royal Perth Hospital, Perth, AUS; 2 Neuroradiology, Neurological Intervention and Imaging Service of Western Australia, Sir Charles Gairdner Hospital, Perth, AUS; 3 Neurosciences, Perron Institute for Neurological and Translational Science, Perth, AUS

**Keywords:** area postrema syndrome, giant cell arteritis, ischaemic stroke, lateral medulla, vertebral artery

## Abstract

A 76-year-old man presenting with intractable nausea, vomiting and hiccoughs was found to have multiple watershed cerebellar strokes and a medullary stroke involving the area postrema. Imaging revealed extensive segmental stenoses involving the bilateral V2-V3 portions of the vertebral arteries. Mildly elevated inflammatory markers raised the possibility of giant cell arteritis (GCA). While the patient exhibited multiple neurological deficits, it was his intractable nausea, vomiting and hiccoughs - manifestations of area postrema syndrome (APS) - that prolonged his admission to a total of 81 days, limiting his ability to engage with rehabilitation. The diagnosis was confirmed with positron emission tomography (PET) imaging. Treatment with corticosteroids was initiated, followed by the introduction of weekly subcutaneous tocilizumab, an IL-6 inhibitor, ultimately resolving both his APS and its underlying cause, giant cell arteritis. To our limited knowledge, this is only the second case of area postrema syndrome caused by GCA in the literature.

## Introduction

Giant cell arteritis (GCA) is a granulomatous large vessel vasculitis predominantly affecting individuals over the age of 50. Granulomatous tissue forms in the arterial media, damaging the internal elastic lamina. GCA typically affects branches of the external carotid artery, posterior ciliary arteries, subclavian arteries, and aortic arch. The classical presentation includes headache, transient visual loss, jaw claudication and elevated inflammatory markers [1]. Stroke is a rare complication of GCA (3-4% of cases), with a marked predilection for the posterior circulation, particularly the vertebrobasilar system [2].

Area postrema syndrome (APS) is a result of injury, often inflammatory or ischaemic, to the area postrema, located on the floor of the fourth ventricle, which is mainly supplied by the posterior inferior cerebellar artery (PICA), a branch of the vertebral artery. Its classical presentation - nausea, vomiting and hiccoughs - is seen in neuromyelitis optica, glial fibrillary acidic protein and myelin oligodendrocyte antibody-associated disease (MOGAD), but it is rarely reported in cerebrovascular disease, and only once in GCA [3].

We discuss a case of stroke, followed by severe APS attributed to vertebral artery vasculitis. This unique GCA presentation highlights the need to broaden the differential diagnosis for causes of APS.

We also explore the pathophysiology of stroke in GCA, which primarily involves stenosis and occlusion, with a particular focus on an insult to the Area Postrema.

## Case presentation

A 76-year-old man presented with acute vertigo worsening over two days, preceded by six months of intermittent vertigo and occipital headaches. Two days before admission, his headache intensified, and his intermittent vertigo became constant, worsening with leftward movement, compromising his ability to mobilise due to extreme nausea. He denied jaw claudication, scalp tenderness and visual disturbance.

Past medical history was significant for well-controlled type 2 diabetes mellitus diagnosed eight years prior without any features of microvascular, autonomic or neuropathic complications, managed with longstanding metformin (extended-release) 500 mg once daily, which was his only regular medication prior to hospitalisation. Otherwise, his past medical history included beta thalassemia with mild anaemia (haemoglobin 110 g/L) and two years of sensorineural hearing loss with tinnitus. He was a never-smoker and drank alcohol rarely.

Cardiorespiratory and abdominal examinations were normal. Neurological examination revealed multiple deficits, including right lateral gaze nystagmus with catch-up saccades on left lateral gaze, left-sided pronator drift, reduced power (4/5) and paraesthesia in the left upper limb, lower limb and face. He demonstrated poor coordination on the left with left-sided past-pointing, a broad-based ataxic gait, and poor proprioception in the left lower limb, causing midline crossing and a lateropulsive gait to the right when walking. Cognitive screening revealed a Rowland Universal Dementia Assessment Scale (RUDAS) score of 26/30, suggesting normal cognition.

**Figure 1:**
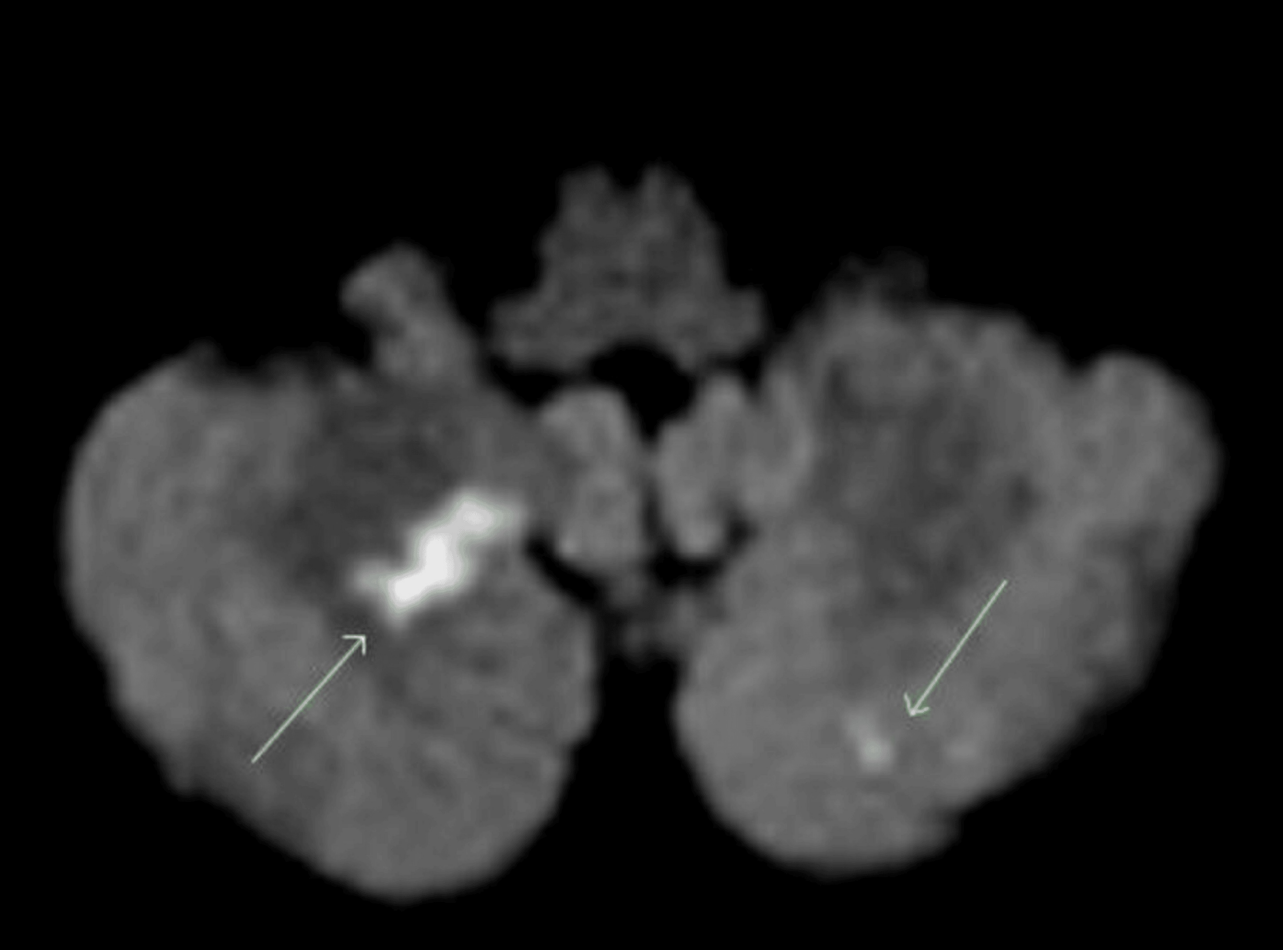
Axial magnetic resonance imaging of the brain demonstrating multiple punctate areas of restricted diffusion consistent with acute infarction in the bilateral cerebellar hemispheres.

**Figure 2:**
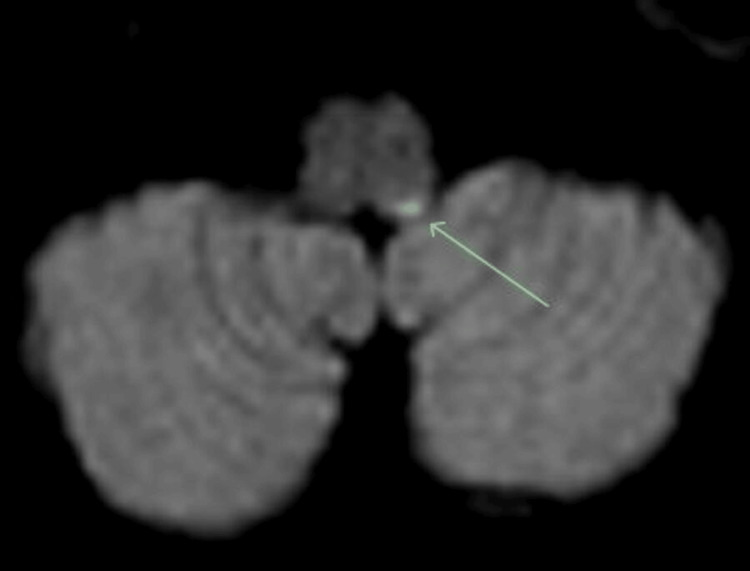
Axial magnetic resonance imaging of the brain demonstrating multiple punctate areas of restricted diffusion consistent with acute infarction in the left hemi-medulla.

**Figure 3:**
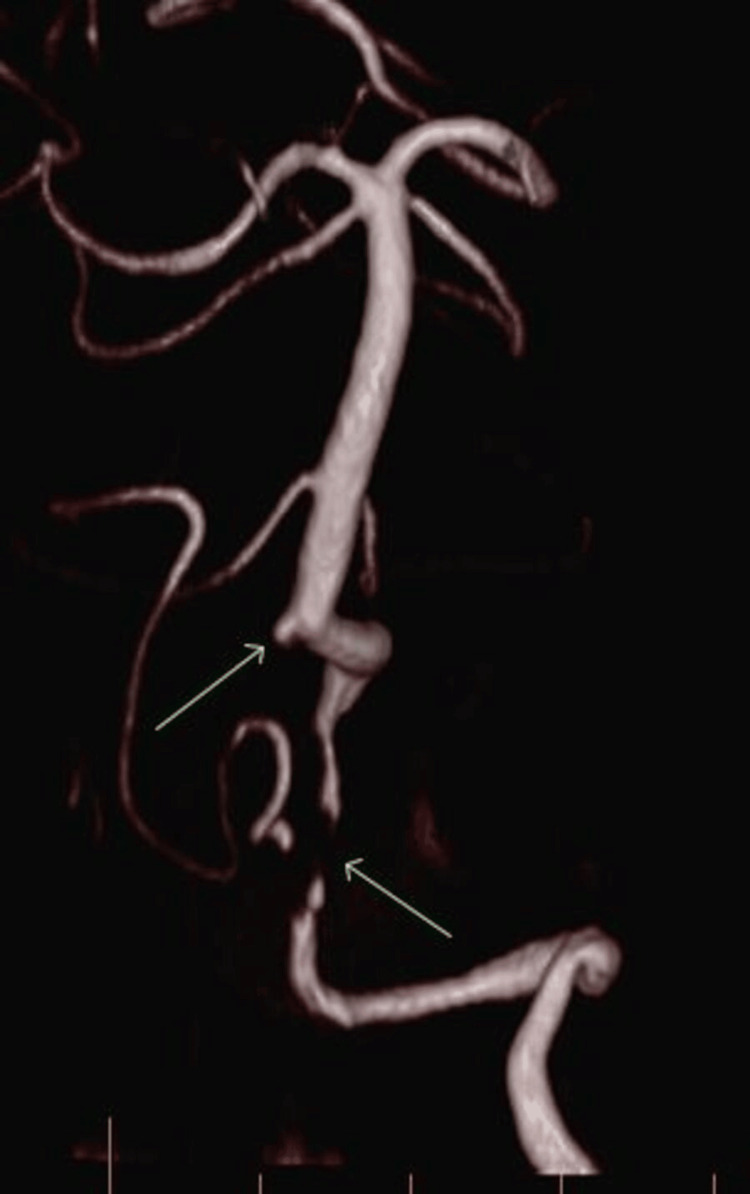
Magnetic resonance angiography of the head and neck demonstrating tight stenosis of the left vertebral artery and near-occlusion of the right vertebral artery, with only intermittent flow visualised. Extensive atherosclerotic changes evident in the cavernous and clinoid segments of the internal carotid arteries, with critical stenosis on the right.

**Figure 4:**
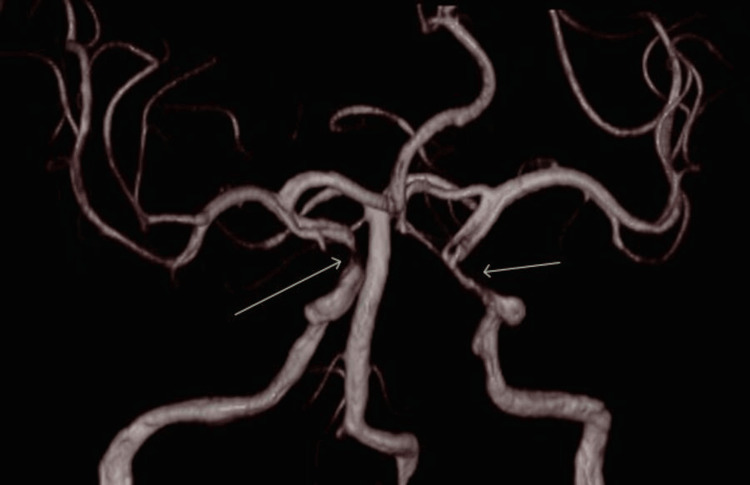
Magnetic resonance angiography of the head and neck demonstrating tight stenosis of the left vertebral artery and near-occlusion of the right vertebral artery, with only intermittent flow visualised. Extensive atherosclerotic changes evident in the cavernous and clinoid segments of the internal carotid arteries, with critical stenosis on the right.

His initial CT brain revealed no haemorrhage. An MRI brain the next day demonstrated multiple bilateral cerebellar and left hemi-medullar punctate acute infarcts, tight stenosis in the left vertebral artery and only intermittent flow in the right vertebral artery, which was essentially occluded. Extensive atherosclerosis was demonstrated in the cavernous and clinoid segment of the internal carotid arteries (ICAs), with tight stenosis on the right (Figures 1-4).

An echocardiogram and Holter monitor result were normal. Biochemical results were normal except for a minimally elevated glycated haemoglobin (HbA1c) of 7.1% (Table 1) and FBC findings consistent with a known history of beta thalassaemia (Table 2).

**Table 1:** Glycated Haemoglobin (HbA1c) Values and Diagnostic Reference Ranges

Test	Result	Reference Ranges
Glycated Haemoglobin (HbA1c)	7.1%	<5.7% (normal) 5.7-6.4% (pre-diabetes) ≥6.5% (diabetes)

**Table 2:** Full Blood Count (FBC) Values and Diagnostic Reference Ranges

Parameter	Result	Reference/Comment
Haemoglobin (Hb)	101 g/L	Low
Haematocrit (Hct)	0.33 L/L	Low
Red Blood Cell Count (RBC)	5.7 x 10^6^/µL	High-normal
Mean Corpuscular Volume (MCV)	63 fL	Low
Mean Corpuscular Haemoglobin (MCH)	19 pg	Low
Mean Corpuscular Haemoglobin Concentration (MCHC)	309 g/L	Low-normal
Red Cell Distribution Width (RDW)	Normal-mildly raised	11.5-14.5%
White Blood Cell Count (WCC)	6.6 x 10^9^/L	Normal
Platelet Count	285 x 10^9^/L	Normal

Aspirin and 80 mg of atorvastatin were commenced, and once the MRI results returned, he was placed on clopidogrel also for dual antiplatelet therapy.

His case was discussed at the neuroradiology meeting. While some atheroma was noted, the patient’s well-controlled Type 2 Diabetes Mellitus, and Ethiopian ethnicity (which does increase the risk of intracranial atherosclerosis) [4] could not explain the burden of disease on imaging. His right vertebral artery did show atypical concentric thickening and raised concerns for vasculitis. The patient’s C-reactive protein (CRP) and erythrocyte sedimentation rate (ESR) were minimally elevated (9.3 mg/L and 38 mm/hr, respectively) (Table 3).

**Table 3:** Inflammatory Markers: C-Reactive Protein (CRP) and Erythrocyte Sedimentation Rate (ESR) Results with Reference Ranges

Test	Result	Reference Range
C-reactive protein (CRP)	9.3 mg/L	<5 mg/L
Erythrocyte sedimentation rate (ESR)	38 mm/hr	0–20 mm/hr

Bilateral temporal artery ultrasound showed concentric and eccentric wall thickening overall favouring atherosclerosis rather than vasculitis; therefore, biopsy was not pursued. Ophthalmology ruled out anterior ischemic optic neuropathy (AION). An autoimmune screen was ordered including aquaporin-4 antibody (AQP4), myelin oligodendrocyte glycoprotein (MOG) antibody testing, anti-neutrophil cytoplasmic antibodies (ANCA), antinuclear antibody (ANA), double-stranded deoxyribonucleic acid antibody (DSDNA), anti-Sjogren’s Syndrome-related Antigen A antibody (SSA) and Anti-Sjogren’s Syndrome-related Antigen B antibody (SSAB), all of which were normal. A fluorodeoxyglucose positron emission tomography (FDG-PET) scan was requested, but due to limited resources, took several days before it was performed at another hospital.

Due to ongoing nausea and vomiting (N&V) despite regular intravenous ondansetron and metoclopramide for two days, the palliative care team adjusted the patient’s antiemetics to metoclopramide, cyclizine and prochlorperazine, providing brief relief only. The patient’s vomiting and vertigo continued to worsen but now with associated hiccoughs. Palliative care suggested trialling gabapentin 100 mg three times daily if the hiccoughs became distressing, but it was not felt to be required as the hiccoughs slowly abated over three days. Conversely, his nausea and vomiting progressed at up to eight episodes of emesis daily.

A nasogastric tube was placed for feeding. Palliative care then suggested regular levomepromazine 6.25 mg SC twice daily with PRN ondansetron, discontinuing metoclopramide and cyclizine. Regular low-dose diazepam was also initiated due to its weak evidence for helping refractory N&V [5].

**Figure 5:**
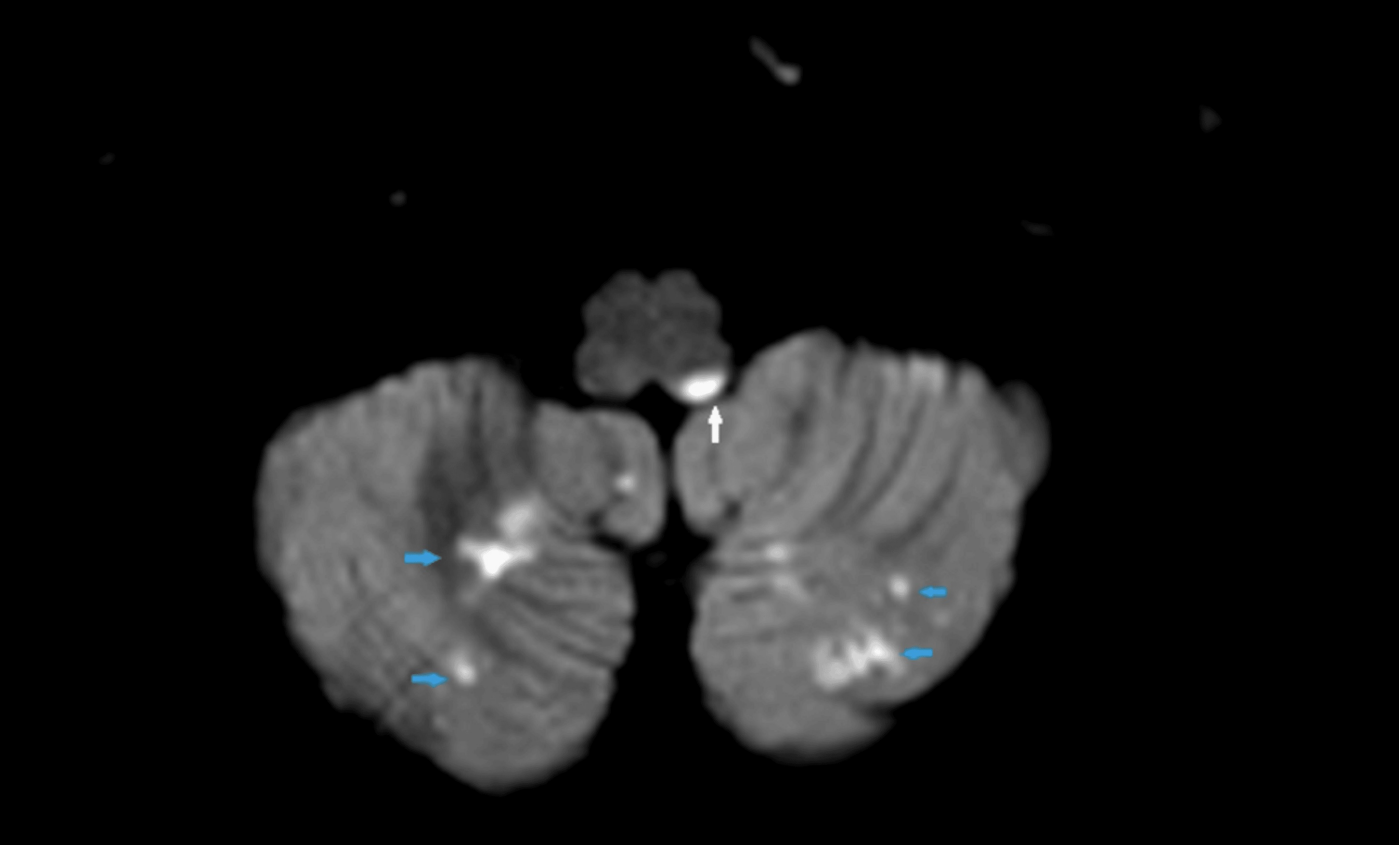
Repeat axial DWI magnetic resonance imaging of the brain performed 12 days after initial imaging shows progression of the cerebrovascular disease despite dual antiplatelet and statin therapy. There is enlargement of the left posterolateral medullary infarct with new acute infarcts identified in the left lateral cervico-medullary junction and left inferior cerebellum. DWI: Diffusion-weighted imaging

A repeat MRI revealed progression of cerebrovascular disease despite dual antiplatelet and statin therapy, with enlargement of the left posterior lateral medullary infarct and new left lateral cervico-medullary junction and left inferior cerebellar infarcts since the MRI performed 12 days prior (Figure 5). There was near complete occlusion of the left intradural vertebral artery at the PICA origin with concentric wall enhancement of the intradural left vertebral artery, along with non-specific right vertebral artery enhancement and T1 hyperintensity in the cervical vertebral artery, suggesting intramural methaemoglobin, possibly indicating prior dissection or vasculitis.

**Figure 6:**
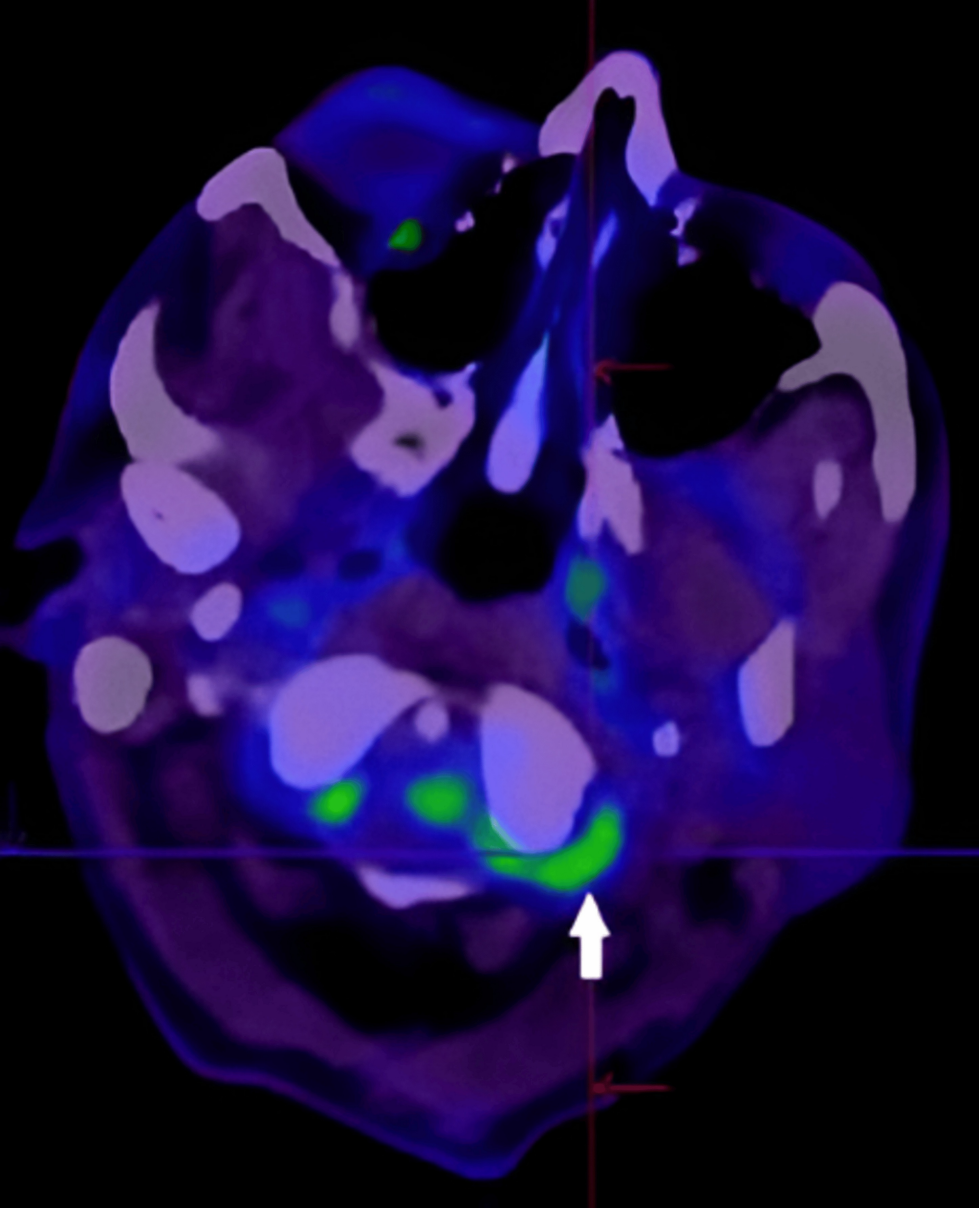
Fluorodeoxyglucose positron emission tomography (FDG-PET) scan demonstrating focal diffuse increased FDG uptake in the high cervical segment of the right vertebral artery, in keeping with active vascular inflammation.

**Figure 7:**
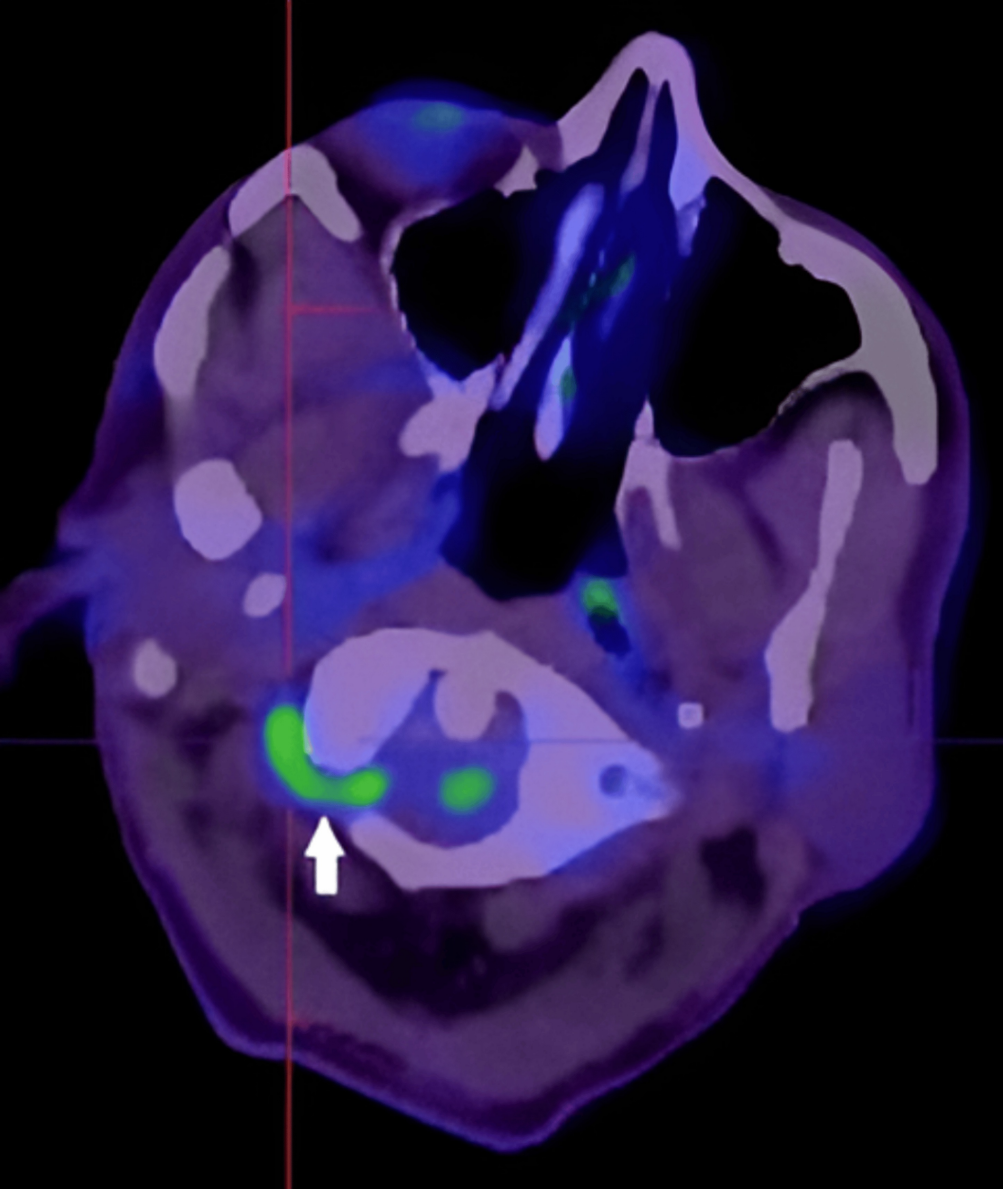
Fluorodeoxyglucose positron emission tomography (FDG-PET) scan demonstrating diffuse increased FDG uptake in the high cervical segment of the left vertebral artery, consistent with active vascular inflammation.

**Figure 8:**
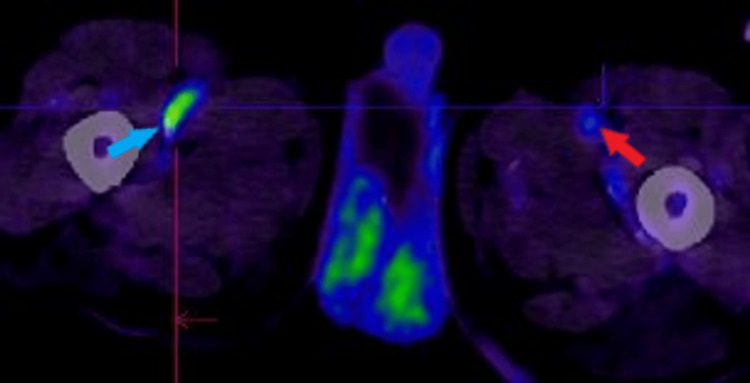
Fluorodeoxyglucose positron emission tomography (FDG-PET) scan demonstrating increased FDG uptake in the proximal right femoral artery, suggestive of active vascular inflammation.

The next day, an FDG-PET scan confirmed active vasculitis involving the bilateral vertebral and right proximal femoral arteries (Figures 6-8), along with signs of a seemingly subclinical concurrent polymyalgia rheumatica.

He received five days of IV methylprednisolone 1g, along with prophylactic calcium 1g once daily, vitamin D 25mcg once daily, co-trimoxazole 80/400mg once daily, and pantoprazole 40mg once daily. Afterwards, he was switched to oral prednisolone at 50mg for two weeks, with a gradual taper. Subcutaneous tocilizumab 162mg once weekly was initiated 15 days after the first methylprednisolone dose, following normal hepatitis B, hepatitis C, HIV, and QuantiFERON-TB tests. During the IV methylprednisolone pulsing, he developed occipital headaches radiating to the bifrontal scalp, and new jaw claudication when eating, but symptoms improved after nine days of treatment. His nausea persisted for six more weeks before completely resolving, and he was discharged from rehabilitation after two months in the hospital.

## Discussion

Area postrema

The area postrema, a circumventricular organ located on the floor of the fourth ventricle in the medulla, is one of the most highly vascularised regions in the brain and lacks a blood-brain barrier (BBB). Its fenestrated capillaries (i.e., “holes”) allow the chemoreceptor trigger zone (CTZ) in the area postrema to detect emetogenic toxins in the blood, triggering the emetic reflex via communication with the nucleus tractus solitarius (NTS) [6].

Area postrema syndrome (APS), characterised by intractable nausea, vomiting and hiccoughs, can rarely be caused by ischaemic stroke, and APS caused by GCA is even rarer. Nausea and vomiting is common in larger strokes, or strokes affecting the posterior fossa, but persistent hiccoughs with severe vomiting suggest a localised issue, particularly involving the area postrema [7].

Stroke and APS

In a cohort of 2,912 stroke patients, 28% had posterior fossa strokes, and 3.7% had medullary strokes, with 88% of medullary strokes being lateral medullary and 12% medial medullary [8]. Stroke is a rare cause of APS with only two cases reported in the literature. Cohen et al. reported a 67-year-old diabetic patient with cerebrovascular and peripheral vascular disease who sustained a cerebellar stroke extending into the medulla and area postrema, resulting in persistent nausea, vomiting and hiccoughs, leading to the diagnosis of an APS [9]. Na et al. described severe intractable APS following a lateral medullary stroke with clear extension into the AP. Symptoms persisted despite the resolution of nystagmus, excluding vestibular stimulation as a cause. APS symptoms resolved after seven days of intensive treatment with metoclopramide, ondansetron, domperidone and itopride [10]. Our patient had a more severe, intractable course, resolving on day 74, following corticosteroid treatment.

The AP is situated in the dorsal/medial medulla, within the territories of both the posterior cerebellar (PICA) and anterior spinal arteries [11]. The rarity of APS with ischaemic strokes may be due to the infrequent involvement of the anterior spinal artery, which despite originating from the V4 segment of the vertebral artery, extensively anastomoses with other cervical feeding vessels [12]. Infarction of the AP requires both the PICA and the anterior spinal artery to be diseased due to its dual blood supply. Lateral medullary syndrome contains elements of the APS with nausea and hiccoughs, but this is often transient and relates to asymmetrical vestibular stimulation, which Na excluded in their case [10]. There is one report of GCA causing lateral medullary syndrome [13]. Infarction of the dorsomedial medulla with a lateral medullary syndrome is uncommon, but radiological extension into the AP is rare and accounts for the rarity of APS in stroke. Na et al. demonstrated unilateral infarction of the AP [10], while our case shows a classical lateral medullary infarct near the midline, not involving the AP. It is likely the connections of the AP were involved, similar to Vlaskovic et al.’s case [11].

Stroke and GCA

GCA is a recognised rare cause of stroke, with incidence varying from 3-11% in stroke cohorts. A French study of GCA found that the risk factors for stroke were male sex, lack of anaemia, polymyalgia rheumatica, and GCA vertebral artery involvement [2].

The diagnosis may be overlooked if GCA symptoms and inflammatory markers are not assessed. Visual loss in GCA is typically immediate and irreversible, making early diagnosis crucial [14]. Only 4% of GCA patients have both ESR and CRP within the normal range. CRP is more sensitive, but elevated ESR with normal CRP can occur [15,16], so testing both is important. In our patient’s case, his inflammatory markers were minimally elevated or entirely normal at times.

The definitions of a GCA-related stroke are: (1) meeting the criteria of the American College of Rheumatology (ACR), and (2) an ischemic stroke occurring at the time of vasculitis diagnosis or within four weeks of starting GCA treatment.

The 1990 ACR/EULAR criteria primarily emphasize cranial features of GCA [17] and are not effective in classifying patients with diseases primarily involving the larger arteries, exhibiting a sensitivity of just 37.1%. The 2022 update includes large vessels due to the poor sensitivity of the previous criteria. The new criteria, using advanced imaging, demonstrate excellent specificity and sensitivity in a large international GCA cohort, with a sensitivity of 87% [18].

Imaging modalities for diagnosing large vessel GCA include CT/CT with angiography, MRI/MR angiography and FDG PET - all three of which our patient underwent. Due to a lack of prospective studies, MRA cannot be recommended over CTA or vice versa [19-22].

Stroke due to GCA has two notable clinical features: Vertebrobasilar involvement is frequent, and intracranial involvement is rare.

Most GCA strokes affect the posterior circulation (70%) [23], presenting ataxia, vertigo, dysarthria, and visual field defects. In large stroke cohorts, screening for GCA using raised inflammatory markers and imaging (temporal and vertebral artery ultrasound) demonstrated an incidence of 2.4-2.8%. Vertebrobasilar strokes account for 15-20% of all strokes and the prevalence of GCA is estimated to be 3.1%. Headache occurred in 60% and most had multiple vertebral artery occlusions or stenoses in all four arterial segments [9]. In a single neurology centre, of 77 cases of GCA cases admitted within a 10-year period, 29 (37%) had vertebral artery involvement on imaging. Stroke was detected in 11 of the 29. In this group, there was a female preponderance and with a lower ESR (62 vs 42 mm/min). Imaging detected stenosis or occlusion of the vertebral artery by ultrasound, CT, or MRI angiography. FDG-PET scan was abnormal in 50% of vertebral arteries [23].

Stroke related to GCA has a poor prognosis with a mortality rate of 14-28% [2]. Intracranial vasculitis is rare because GCA mainly affects arteries with elastic tissue, which intracranial arteries lack [24]. The mechanisms are occlusion from vascular inflammation or stenosis with a watershed rather than thrombosis [25].

Intimal proliferation, narrowing the arterial lumen, may explain the higher incidence of stroke in vertebral artery territories versus the carotid arteries, which are therefore vulnerable to haemodynamic change. Despite this, aspirin is recommended, though studies show inconsistent results in reducing ischaemic events. There are cases of GCA and carotid and vertebral artery dissection [26]. The strokes occur at presentation or in the four weeks following initiation of therapy.

When the carotid artery is involved, the siphon is the most frequently involved segment [27,28]. Oerding et al. performed a literature search detailing 39 cases of symptomatic ICA GCA, noting characteristic bilateral distal ICA involvement (cavernous and para-clinoid segments), with terminal ICA stenosis as a long-term consequence [29].

## Conclusions

In summary, our patient presented with a lateral medullary infarction and subsequently developed severe area postrema syndrome (APS). Further evaluation revealed large vessel vasculitis consistent with giant cell arteritis (GCA), diagnosed through advanced imaging, specifically fluorodeoxyglucose positron emission tomography (FDG-PET). This case highlights the importance of considering GCA in the differential diagnosis of APS. Supporting features include elevated inflammatory markers and targeted vascular imaging - particularly of the vertebral arteries - as the area postrema receives a dual blood supply from the posterior inferior cerebellar artery (PICA) and the anterior spinal artery, both of which arise from the vertebral circulation. To our knowledge, our patient represents only the second reported case of APS secondary to GCA, and the third associated with a posterior fossa infarction.
